# Radiation-induced osteosarcoma of the skull base after radiation therapy in a patient with nasopharyngeal carcinoma: a case report and review of the literature

**DOI:** 10.1186/s13256-016-1112-3

**Published:** 2016-12-01

**Authors:** Yassine Echchikhi, Hasna Loughlimi, Asmae Touil, Tayeb Kebdani, Noureddine Benjaafar

**Affiliations:** Department of Radiation Oncology, National Institute of Oncology, University Mohamed 5, Ibn Sina Center, Allal El Fassi Boulevard, Rabat, Morocco

**Keywords:** Osteosarcoma, Skull, Radiotherapy, Nasopharynx, Chemotherapy, Pathogenesis

## Abstract

**Background:**

Radiation-induced osteosarcomas are a recognized complication of radiation therapy. Owing to the fact that it is rare, publications on radiation-induced osteosarcoma of the skull base are limited to a small series and some case reports.

**Case presentation:**

We describe a rare case of a patient with a skull base radiation-induced osteosarcoma treated 11 years before with ionizing radiation for an undifferentiated carcinoma of the nasopharynx. The patient was treated with chemotherapy alone, but he died after the third cycle.

**Conclusions:**

Radiation-induced osteosarcoma of the skull base after treatment of nasopharyngeal carcinoma is a very rare but very aggressive complication with a poor prognosis. Chemotherapy gives bad results, and regular follow-up of treated patients should be considered.

## Background

Radiation-induced osteosarcomas are rare clinical entities that are recognized as a complication of radiation therapy and are associated with a poor prognosis. Their incidence ranges from 0.01 % to 0.03 % of all irradiated patients [1, 2] and 5.5 % of all osteosarcomas [2]. This incidence could increase in the future because radiation therapy has become more common and developed, and patient survival has improved. Reports of radiation-induced osteosarcoma of the skull base are limited to a small series and some case reports. In this report, we describe a very rare case of skull base radiation-induced osteosarcoma in a patient treated with ionizing radiation for undifferentiated carcinoma of the nasopharynx.

## Case presentation

A 29-year-old Moroccan man presented to our hospital with a 6-month history of headache in his left skull, associated with homolateral facial pain, numbness, diplopia, exophthalmia, eye watering, and an episode of epistaxis. Eleven years before, he had received radiotherapy for undifferentiated carcinoma of the nasopharynx, initially classified as T1N1M0. The radiation dose he had received was 70 Gy in 35 fractions delivered using the classic 3-fields technique (2 lateral opposed fields abutted to an anterior low-neck field). This technique had been applied during the previous 20 years because it seemed to be the simplest.

**Fig. 1:**
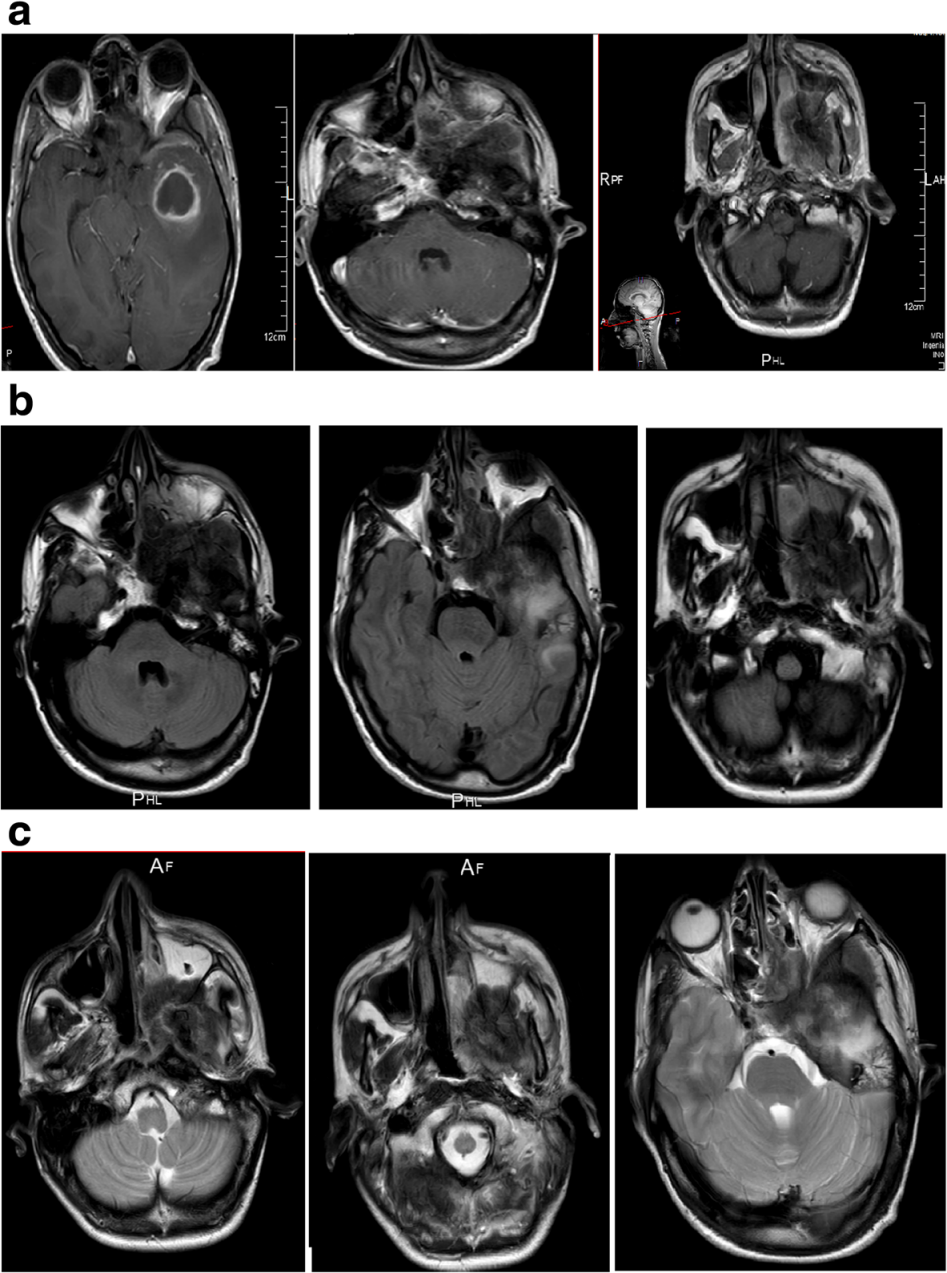
Magnetic resonance imaging studies show a tumor process of the skull base involving the sphenoid bone with its two left wings, the squamous part of the left temporal bone, associated with intracranial expansion, as well as a second, isolated mass in the temporal lobe. **a** T1-weighted, gadolinium-enhanced axial magnetic resonance imaging scans. **b** T1-weighted, axial magnetic resonance imaging scans. **c** T2-weighted axial magnetic resonance imaging scans

The patient’s physical examination revealed a left ptosis, hemifacial edema, and decreased visual acuity. Magnetic resonance imaging disclosed a tumor process of the skull base involving the sphenoid bone with its two left wings, the squamous part of the left temporal bone, and the left maxillary bone, associated with intracranial expansion, as well as a second, isolated mass in the temporal lobe (Fig. 1).

**Fig. 2:**
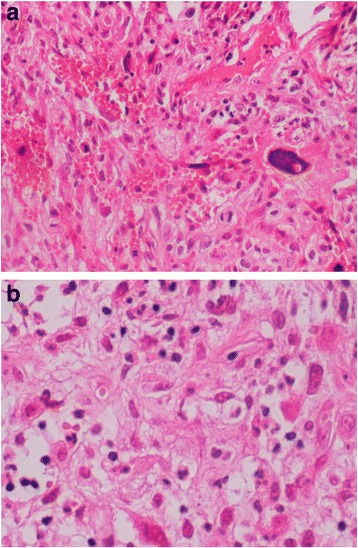
**a** and **b** High-grade osteosarcoma with spindle-cell pleomorphic neoplasm, badly limited cytoplasm, and osteoid matrix. Original magnification × 20 (**a**) and × 40 (**b**)

Direct nasofibroscopy with biopsy was performed. The histological results with immunohistochemistry were in favor of osteosarcoma with the following characteristics: spindle-cell neoplasm pleomorphism with badly limited cytoplasm, an irregular nucleus with frequent atypical mitoses, and an associated osteoid matrix. The results of immunohistochemistry with anti-AE1/AE3, anti-endomysial antibodies, and anti-CD34/CD31 were negative (Fig. 2).

A complete search for distant metastasis by positron emission tomography and by enhanced computed tomography of the chest, abdomen, and pelvis produced no results. We reviewed the initial pathology and documentation outlining the patient’s original radiation therapy and confirmed that the tumor developed in the previously irradiated field. The tumor was deemed unresectable, and further radiation therapy was not advised. The patient was started on chemotherapy consisting of four cycles of ifosfamide, cisplatin, and doxorubicin, to be followed by four planned cycles of doxorubicin and ifosfamide. A physical evaluation after the second cycle revealed improvement of clinical signs, but after this initial remission, the patient’s condition deteriorated, and he died 1 month after his third cycle of chemotherapy.

## Discussion

Radiation therapy for head and neck cancer is a well-accepted modality of treatment. It is a standard treatment for carcinoma of the nasopharynx. However, as with all treatment modalities, there are short- and long-term morbidities and occasionally mortalities. One of the most dreaded complications of radiation therapy is the formation of new malignancies. Although difficult to estimate, the incidence of radiation-induced tumors is extremely low [3]. The most frequent radiation-induced tumors are fibrosarcoma and osteosarcoma. Sarcomatous tumors account for most radiation-induced neoplasms involving the head and neck, and most occur in the facial skeleton. Very few involve the skull base [4–7]. Postirradiation osteosarcomas are rare, accounting for 3–6 % of all cases of osteosarcoma and 0.01–0.03 % of all irradiated patients [1, 2]. The incidence of radiation-induced tumors is increasing in the oncology population as a result of increased survival through the development of cancer treatment [8]. Cahan *et al.* described the following criteria for radiation-induced sarcoma: (1) The initial and secondary neoplasms are of significantly different histological types; (2) the secondary neoplasm must arise within the irradiated area; (3) there must be a long latency period after radiation (>5 years); and (4) all sarcomas must be proven histologically [9]. Our patient fulfilled all these conditions, and the diagnosis of radiation-induced osteosarcoma was thus established.

The pathogenesis of postirradiation sarcoma is unknown. Various predisposing factors have been suggested: radiation dose, age of the patient at radiation exposure, association with chemotherapy, and genetic predisposition [10]. Documentation of new mutations in genes implicated in malignant transformation after radiation therapy, such as the *p53* and retinoblastoma tumor suppressor genes, further supports induction of malignancy [11], suggesting that loss of these tumor suppressor genes plays a role in the development of these lesions [12, 13]. Friend *et al.* proposed a link between retinoblastomas and osteosarcomas [14], and Patel *et al.* reported 2 of 16 patients who had retinoblastoma as the original lesion, for which the patients received radiation [15].

The latency period specified by Cahan *et al.* is more than 5 years. Since publication of their report, there have been some reports of patients developing radiation-induced sarcomas with shorter latency periods (as short as 2 years) [15–18]. Laskin *et al.* [19] reported a link between shorter latency periods and worse outcomes with radiation-induced soft tissue sarcomas, but this relationship has not been further substantiated in the literature. Patel *et al.* did not find any significant correlation between latency period and survival [15]. It is believed that the mean latency is inversely proportional to the dose received in radiotherapy. However, some authors indicate that the inverse relationship between dose and latency applies only to very high doses [20–22]. Other authors have noted that the latency period ranged from 3.5 to 33 years (median 10 years) [23]. In our patient, the tumor appeared 11 years after radiation.

Our review of skull base radiation-induced osteosarcoma cases revealed that the most common primary tumor for which radiation was initially received is pituitary adenoma. We found no case of radiation-induced osteosarcoma in skull base after radiotherapy for nasopharyngeal carcinoma. Otherwise, the anatomic site of the sarcoma has important implications for treatment and outcome. Reports detailing radiation-induced sarcoma in patients who were treated for head and neck cancer, particularly for nasopharyngeal carcinoma, are relatively few and limited to case reports and a few small case series [6, 24, 25].

Mian *et al.* reported a series of 53 patients who developed radiation-induced malignancies after radiotherapy for nasopharyngeal carcinoma between 1964 and 2003. Fibrosarcoma was the most frequent histologic type at 41.5 % (22 cases), followed by osteosarcoma at 22.6 % (12 cases), without any being located in the skull base [25]. Malone *et al.* reported 35 cases of second malignant tumors after treatment of nasopharyngeal carcinoma. Ten cases of osteosarcoma were found, also without any located in the skull base [26]. Radiation-induced sarcoma in patients who were treated for head and neck cancer most often occurs in the facial skeleton; very few involve the skull base [5, 11, 25–27].

Complete surgical excision is the treatment of choice for radiation-induced osteosarcoma, although this can be difficult when there is skull base involvement. The benefit of adjuvant therapy has not been established on a previously irradiated field [8]. For patients with unresectable tumors, radiotherapy and/or chemotherapy are acceptable alternatives. Some reports described the effectiveness of chemotherapy, with variable results, using doxorubicin, ifosfamide, methotrexate, carboplatin, vincristine, or etoposide [28–30], but a definitive protocol has not been established.

Radiation-induced osteosarcomas are considered to be highly aggressive lesions. Local recurrence for primary osteosarcomas of the head and neck has been reported to be 22 % [31], and 86 % for radiation-induced osteosarcoma [15].

Patel *et al*. [15] compared a skull base radiation-induced osteosarcoma group with a calvarial radiation-induced osteosarcoma group and found that the median progression-free survival intervals were 9.5 months and 19 months, respectively (*P* = 0.6322). These differences were not statistically significant. The median overall survival times of patients with radiation-induced osteosarcoma of the skull base was 41 months (Table 1).

**Table 1:** Characteristics of various published cases of skull base radiation-induced osteosarcoma

First author (year) [reference]	Age in years/sex	Primary tumor	Radiation dose (Gy)	Latency (years)	Site of radiation-induced osteosarcoma	Treatment	Survival period
Amine (1976) [32]	16/F	Pituitary adenoma	51.0	10	Sella	Radiation	5 weeks
Tanaka (1989) [33]	57/M	Craniopharyngioma	110	15	Sphenoid wing	Embolization surgery	2 weeks
Salvati (1994) [34]	45/M	Pituitary adenoma	44	12	Sphenoid	Radiation (50 Gy)	16 months
Gnanalingham (2002) [35]	67/F	Pituitary adenoma	52	14	Sella	Surgery	–
Hansen (2003) [36]	39/M	Pituitary adenoma	52	22	Sella, clivus	Surgery	Short
Bembo (2004) [37]	45	Pituitary adenoma	46	5	Sella	Surgery	7 weeks
Patel (2011) [15]	44	Craniopharyngioma	60	9	Sphenoid-ethmoid sinus	Chemotherapy	16 months
Yamada (2012) [38]	75/F	Pituitary adenoma	50	20	Sphenoid sinus	Cyberknife surgery, chemotherapy	24 months
Patel (2011) [15]	54/M	Maxillary and adenoid cystic carcinoma	60	12	Sphenoid, frontal and temporal bone	Surgery, chemotherapy	13 months
	63/M	Squamous cell carcinoma, nasal cavity	–	4.5	Palate + maxillary sinus	Surgery, chemotherapy	47 months
	50/M	Squamous cell carcinoma, nasal cavity	–	14	Infratemporal fossa	Surgery, chemotherapy	17 years
	44/F	Craniopharyngioma	–	9	Sphenoidal, ethmoidal bone	Chemotherapy	18 months
	15/M	Retinoblastoma	–	15	Zygoma	Surgery, chemotherapy	62 months
	22/M	Retinoblastoma	–	20	Ethmoid + maxillary sinus	Surgery, chemotherapy	–
	10/M	Rhabdomyosarcoma	–	6.75	Mastoid + jugular foramen	Surgery, chemotherapy, radiation	29 months
	31/M	Rhabdomyosarcoma	60	18	Palate + infratemporal fossa	Surgery, chemotherapy	143 months
	26/M	Retinoblastoma	35	25		Surgery, chemotherapy	41 months
Hansen (2003) [36]	29/M	Pituitary adenoma	5000 rad	22	Sphenoid, frontal clivus	Chemotherapy	Very short
Patel (2014) [39]	52/M	Craniopharyngioma	50	22	Sella + clivus	Surgery	1 month
Our patient	28/M	Nasopharynx carcinoma	70	11	Sphenoid + maxillary bone	Chemotherapy	6 months

*M* male, *F* female, *Gy* gray

The 5-year disease-free survival time for patients with radiation-induced osteosarcoma has been reported to be 17 % [10], as opposed to 70 % in patients with primary osteosarcomas of the head and neck [31].

## Conclusions

Radiation-induced osteosarcoma of the skull base after treatment of nasopharyngeal carcinoma is a very rare but very aggressive complication with a poor prognosis. The overall incidence seems quite low and should not change current practice; however, regular follow-up of treated patients should be considered. Complete surgical excision, if possible, can optimize survival of these patients when the tumor is detected at an early stage.
